# Exploring the relationship among soccer-related knowledge, attitude, practice, and self-health in Chinese campus soccer education

**DOI:** 10.1016/j.isci.2024.109409

**Published:** 2024-03-04

**Authors:** Honglin Song, Yutao Li, Zhenhang Zhang, Tianbiao Liu

**Affiliations:** 1 College of Physical Education and Sports Science, Beijing Normal University, Beijing 100875, China

**Keywords:** health sciences, sports medicine, Natural sciences

## Abstract

China has promoted campus soccer for over a decade due to its potential health benefits. The study aimed to explore soccer knowledge (SK), soccer attitude (SA), soccer practice (SP), and health status among Chinese freshmen and sophomore undergraduates who had received campus soccer education. Of the 7419 participants, 1,069 were valid and included in the analysis. Structural equation modeling (SEM) results indicated SK is positively associated with SA (p < 0.001), but negatively with SP (p < 0.01). SA was positively linked to SP (p < 0.001). SK indirectly affected SP through SA (Z = 13.677). Random forest-tree-structured Parzen estimators (RF-TPE) with SHAP indicated SP holds primary importance with a strong negative impact on health. Additionally, differences in rankings for SK, SA, and SP were observed among gender and urban-rural groups. These results reveal current campus soccer education is suboptimal to health promotion.

## Highlights

• This study explored the current Chinese campus soccer education situation
• The findings can provide implications for future campus soccer education

## Graphical abstract

**Figure:**
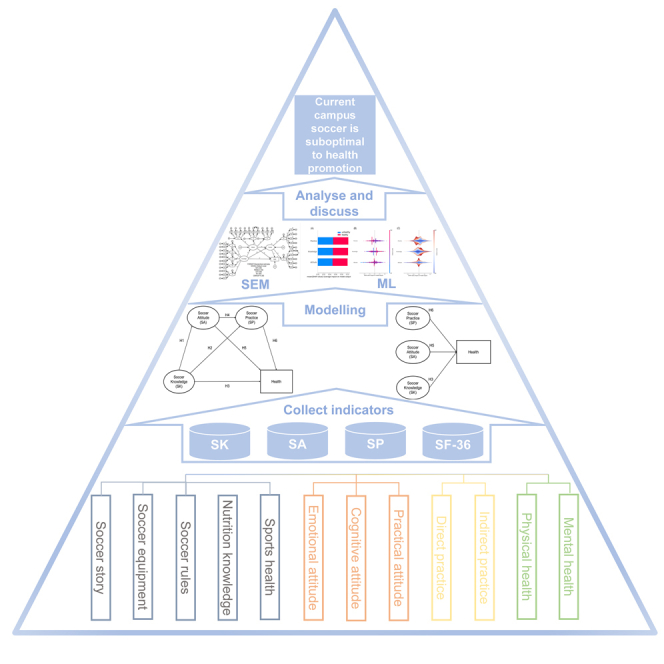

## Introduction

Campus soccer teaching interventions are believed to reduce blood pressure, improve cardiovascular function, increase muscle strength, and lower body fat, thereby enhancing adolescents’ physical functions.^1^^,^^2^ Moreover, regular soccer exercise is thought to boost adolescents’ confidence, self-efficacy, sense of achievement, and self-expression,^3^ consequently improving their mental health.^4^ Considering the potential health benefits of soccer exercise, campus soccer has been vigorously developed in China and has been implemented for more than 10 years now. In order to ensure the orderly and scientific development of campus soccer, relevant studies have emerged in recent years. These studies include the following four areas: (1) evaluating the construction of characteristic schools for campus soccer,^5^^,^^6^^,^^7^ (2) investigating the current state of the training and competition system,^8^^,^^9^^,^^10^^,^^11^^,^^12^ (3) exploring whether campus soccer promotes the physical and mental health of youth,^13^^,^^14^^,^^15^ and (4) drawing on the experience of developed soccer countries in youth soccer development.^16^^,^^17^ These findings are expected to propel campus soccer into a more critical development stage, enhance students’ soccer skills, promote the physical and mental health of adolescents, and offer decision-makers more scientific and effective management strategies for campus soccer.

Previous studies have found that campus soccer meets many challenges, which led to many obstacles to the implementation of soccer in campus. Issues such as unclear tasks, vague goals, and lack of responsibility have emerged in the construction of characteristic soccer schools. In the competition system, there are challenges related to uncoordinated policy development, an inadequate talent supply chain, blockages between campus and professional events, insufficient allocation of competition resource guarantees, and outdated evaluation and supervision of events.^18^^,^^19^

However, two important aspects are overlooked in previous studies. Firstly, previous studies have neglected the essence and the primary purpose of campus soccer: to improve youth physical health. Campus soccer bears the crucial responsibility of enhancing the physical fitness and mental well-being of children and adolescents. However, most studies on campus soccer have focused on cultivating elite soccer talents, neglecting its health-promoting function. While some researchers have confirmed the beneficial effects of campus soccer on the physical and mental health of adolescents through educational interventions, it is essential to note that results obtained in experimental settings may not fully represent the real situation of campus soccer implementation in China. While systematic and scientific soccer education can potentially promote the physical and mental health of adolescents, poorly conducted activities may fail to achieve these benefits and could even lead to harm.^20^^,^^21^

Secondly, previous studies have suffered a dearth of empirical studies on the implementation of campus soccer, indicating a lack of microscopic, quantitative, and in-depth research on the actual execution. These studies focused too much on policy documents and the macro-institutional level for qualitative analysis and discussion, without providing convincing evidence for their views, lacking objective data support, and hindering the widespread application or validation of research conclusions. To address this problem, we advocate for the introduction of quantitative research methods, collecting and analyzing specific statistical data to better understand the details of the current situation and influencing factors of campus soccer implementation from a micro perspective.

Additionally, the knowledge, attitude, and practice (KAP) theory^22^ is considered effective in addressing problems related to educational effectiveness evaluation. KAP theory explains how people’s actions are influenced by their information, emotions, and beliefs about a topic. KAP theory holds that healthy knowledge is the basis for establishing positive attitudes and healthy behaviors, attitudes are the driving force of behavioral change, and promoting healthy behaviors is the goal. In the field of sports^23^^,^^24^^,^^25^ and physical education,^26^^,^^27^ KAP theory has been adopted to examine the effectiveness of injury prevention and health courses in improving athletes’ and students’ health. In this study, KAP theory is adopted to assess the effectiveness of campus soccer education, determining whether it has contributed to the participation and development of youth soccer.

**Figure 1:**
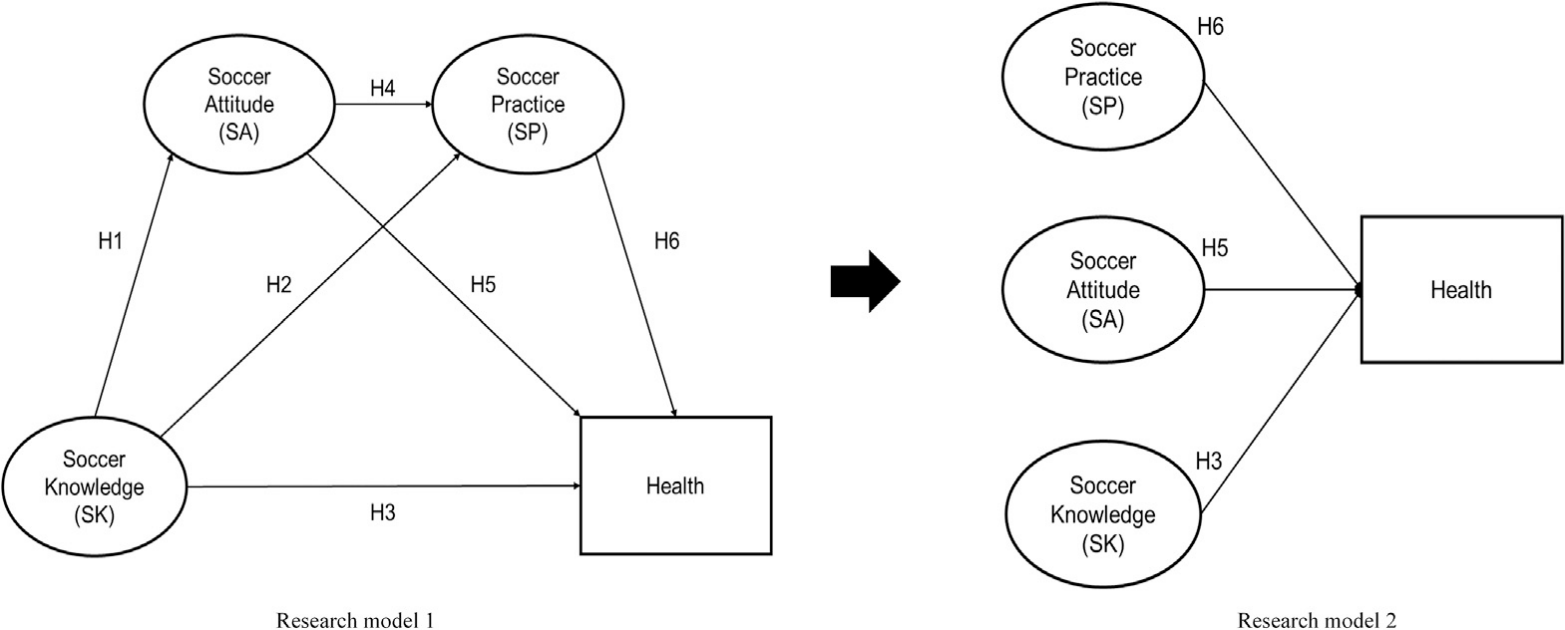
Research models

This study aims to investigate whether campus soccer can follow the KAP theoretical model and the health promotion, i.e., to first develop soccer knowledge, then improve soccer attitudes, and ultimately promote participation in soccer to improve overall physical and mental health. Therefore, this study proposed six hypotheses in the following section and conducted an empirical study based on a survey of 7,040 Chinese students using four scales of soccer knowledge (SK), soccer attitude (SA), soccer practice (SP), and health on their own. To summarize the aforementioned study design, two research models are shown in Figure 1. Structural equation modeling (SEM) and random forest-tree-structured Parzen estimators (RF-TPE) with Shapley additive explanation (SHAP) were applied to assess the associations among study variables. Thus, this study has three aims: (1) to explore the current Chinese campus soccer education situation in terms of SK, SA, SP, and health among students, (2) to explore the relationships among SK, SA, SP, and health, and (3) to investigate the influential factors to provide a more accurate reference for future campus soccer education in China.

H1. Individuals’ soccer knowledge has a significant positive effect on their soccer attitudes.

H2. Individuals’ soccer knowledge has a significant positive effect on their soccer practice.

H3. Individuals’ soccer knowledge has a significant positive effect on their health.

H4. Individuals’ soccer attitudes have a significant positive effect on their soccer practice.

H5. Individuals’ soccer attitudes have a significant positive effect on their health.

H6. Individuals’ soccer practice has a significant positive effect on their health.

## Results

### SEM fit tests

The fit performance of the SEM is an important criterion for evaluating its validity. The better the model fits, the more explanatory validity the model has for data. Based on previous studies,^28^ evaluating model fit involves a rigorous assessment through multiple indicators, each addressing different aspects of fit between the model and the observed data. The chi-square to degrees of freedom ratio gauges the model’s overall discrepancy per degree of freedom, aiming for lower values. The root-mean-square error of approximation considers error approximation per degree of freedom, with lower values indicating a better fit. Comparative indicators like normed fit index, relative fit index, incremental fit index, Tucker-Lewis index, and comparative fit index contrast the proposed model against a baseline model, with values nearing 1 signifying superior fit. Finally, the goodness of fit index and adjusted goodness of fit index reflect the proportion of variance explained by the model, with higher values preferred. Collectively, these indicators provide a multifaceted evaluation of the SEM’s fit, where meeting or exceeding the respective thresholds across these metrics indicates a robust and valid model that adequately represents the observed data. Table 1 demonstrates that these indicators meet the established criteria, indicating that the model exhibits good fit performance.

**Table 1:** Model fit summary for the proposed research model

Index	CMIN/DF	RMSEA	NFI	RFI	IFI	TLI	CFI	GFI	AGIF
Norm	<5	<0.08	>0.90	>0.95	>0.95	>0.95	>0.95	>0.8	>0.8
Value	4.335	0.056	0.960	0.956	0.969	0.965	0.969	0.884	0.863

CMIN/DF, chi-square to degrees of freedom ratio; RMSEA, root-mean-square error of approximation; NFI, normed fit index; RFI, relative fit index; IFI, incremental fit index; TLI, Tucker-Lewis index; CFI, comparative fit index; GFI, goodness of fit index; AGIF, adjusted goodness of fit index.

### Structural results

**Figure 2:**
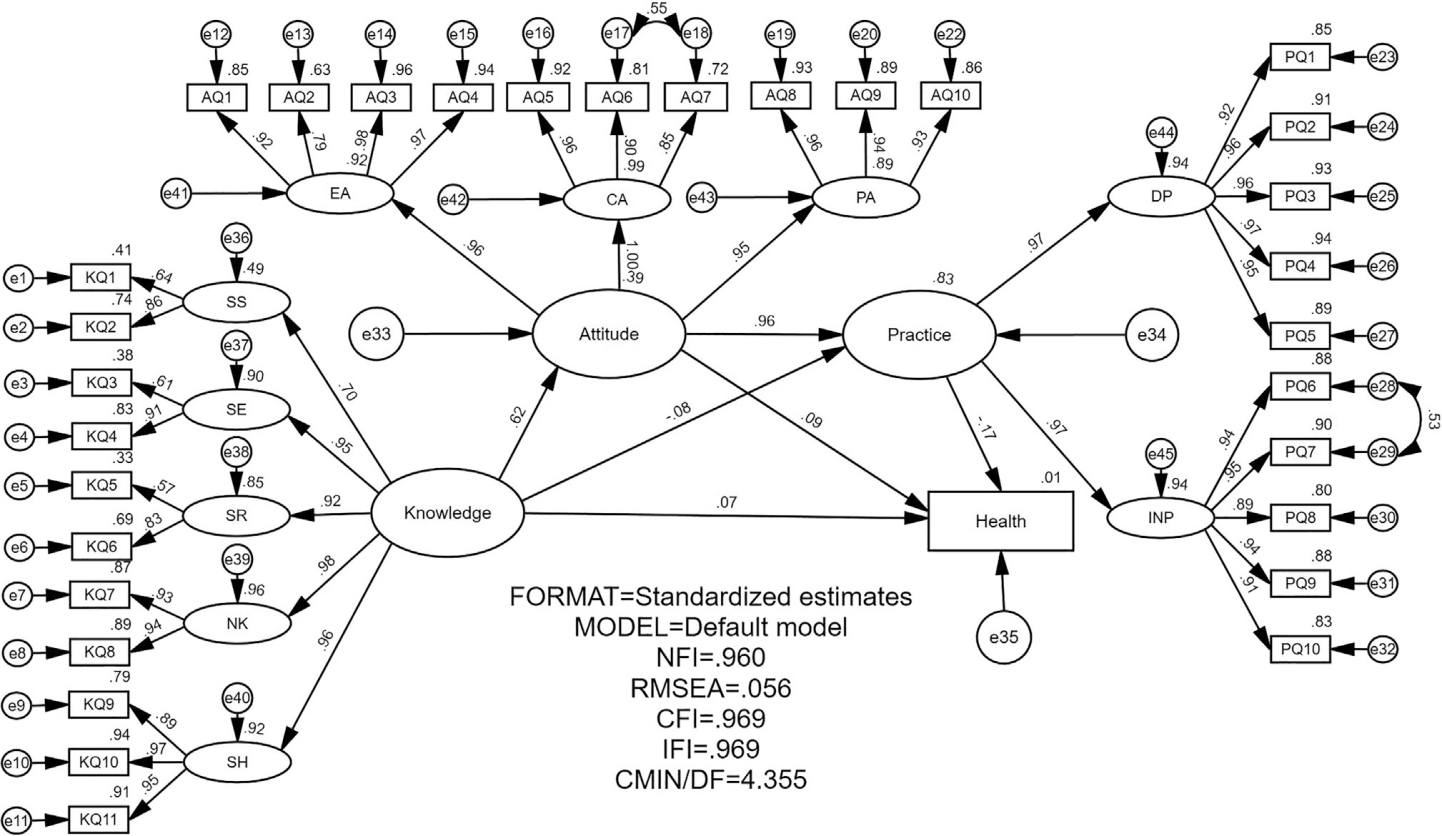
Results of the structural equation model and hypothesis testing CMIN/DF, chi-square to degrees of freedom ratio; RMSEA, root-mean-square error of approximation; NFI, normed fit index; IFI, incremental fit index; CFI, comparative fit index.

Table 2 and Figure 2 show the structural results. The results show that two of the hypotheses proposed in this study are supported, and four hypotheses are rejected. The direct and positive effects of knowledge on attitude are statistically significant (standardized direct effect β = 0.621, p < 0.001), so H1 is supported. The direct and negative effects of knowledge on practice are statistically significant (standardized direct effect β = -0.076, p < 0.001). However, due to the negative path coefficient, H2 is rejected. The direct and positive effects of attitude on practice are statistically significant (standardized direct effect β = 0.957, p < 0.001), so H4 is supported. There are no significant relationships in terms of the direct effect of attitude on health (standardized direct effect β = 0.053, p > 0.05), practice on health (standardized direct effect β = 0.09, p > 0.05) and knowledge on health (standardized direct effect β = 0.065, p > 0.05). Thus, H3, H5 and H6 are rejected.

**Table 2:** Results of the structural equation model and hypothesis testing

Path	Estimate	S.E.	C.R.	P	Hypothesis	Supported
Knowledge→Attitude	0.621	0.047	16.67	∗∗∗	H1	YES
Knowledge→Practice	−0.076	0.030	−3.668	∗∗∗	H2	NO
Knowledge→Health	0.065	0.057	1.561	0.118	H3	NO
Attitude→Practice	0.957	0.032	33.736	∗∗∗	H4	YES
Attitude→Health	0.088	0.106	0.898	0.369	H5	NO
Practice→Health	−0.167	0.086	−1.849	0.064	H6	NO

P, probability value: ∗p < 0.05, ∗∗p < 0.01, ∗∗∗p < 0.001; S.E., standard error; C.R., critical ratio.

### Mediating effect analysis

There are many methods to analyze the mediation effect, such as the causal-step method and the Sobel test. However, previous studies^29^^,^^30^ indicated that mediation effect analysis with bootstrapping is more accurate than the aforementioned two methods. Thus, this study adopted bootstrapping to analyze the mediating variable effects, performing bootstrapping at a 95% confidence interval with 5,000 samples. The asymptotic critical ratio (Z) and the confidence interval of the lower and upper bounds (95% BC, 95% percentile) were used to test whether the indirect effects were significant. When Z > 1.96 and a 95% confidence interval does not contain zero, there is statistical significance. Table 3 shows that the indirect effect of K- > A- > P is statistically significant (Z > 1.96, 95% confidence interval does not contain zero), and the indirect effects of K- > A- > H, K- > A- > P- > H, and K- > P- > H have no significant relationships.

**Table 3:** Results of mediating effect analysis

Effect source	Point estimate	Product of coef		BCEBC 95%			Percentile 95%		
		SE	Z	Lower	Upper	P	Lower	Upper	P
K- > A- > P	0.848	0.062	13.677	0.732	0.973	0.000	0.732	0.974	0.000∗∗∗
K- > A- > H	0.075	0.094	0.798	−0.120	0.249	0.425	−0.122	0.247	0.437
K- > A- > P- > H	−0.135	0.083	−1.627	−0.290	0.037	0.120	−0.289	0.040	0.126
K- > P- > H	0.017	0.013	1.308	−0.002	0.054	0.075	−0.004	0.048	0.129

P, probability value : ∗p < 0.05, ∗∗p < 0.01, ∗∗∗p < 0.001; S.E., standard error; BCEBC, bootstrap confidence intervals for the effect/bootstrap estimates of causal indirect effect.

### Machine learning model performance comparison

Table 4 shows the testing performance of four machine learning models in 10-fold cross-validation. The results show that RF-TPE has the best performance in terms of four key metrics, that is, recall, precision, accuracy, and F1. Thus, it is appropriate to select RF-TPE for further analysis with SHAP.

**Table 4:** The testing performance of four machine learning models in 10-fold cross-validation (mean ± standard deviation)

	Recall	Precision	Accuracy	F1
CatBoost-TPE	0.5593 ± 0.0193	0.5603 ± 0.0200	0.5603 ± 0.0189	0.5567 ± 0.0188
LightGBM-TPE	0.5610 ± 0.0244	0.5620 ± 0.0252	0.5622 ± 0.0238	0.5585 ± 0.0238
RF-TPE	0.5681 ± 0.0340	0.5692 ± 0.0349	0.5659 ± 0.0361	0.5637 ± 0.0357
DT-TPE	0.5429 ± 0.0424	0.5456 ± 0.0424	0.5435 ± 0.0414	0.5345 ± 0.0451

CatBoost, gradient boosting + categorical features; LightGBM, light gradient boosting machine tree; RF, random forest; DT, decision tree; TPE, tree-structured Parzen estimators.

### Global analysis

**Figure 3:**
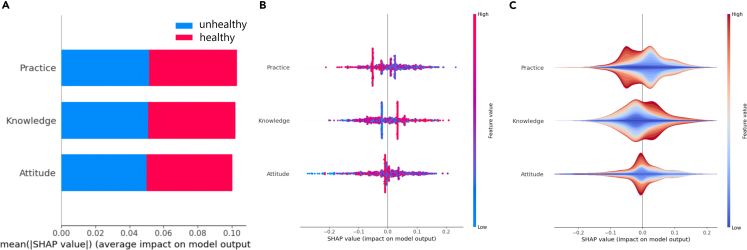
SHAP summary plot related to output (healthy = 1, unhealthy = 0) (A) Bar plot, (B) beeswarm plot and (C) violin plot.

Figure 3A shows SHAP’s global interpretation of the RF-TPE model in all data, and three features are sorted according to their importance, indicating that practice ranks top, followed by knowledge and attitude. As shown in Figures 3B and 3C, the SHAP summary plot provides more detail about the relationship between features and outputs. The result shows that the higher the values of practice, the more likely students are to be unhealthy, indicating that practice has a strong negative impact on health.

**Figure 4:**
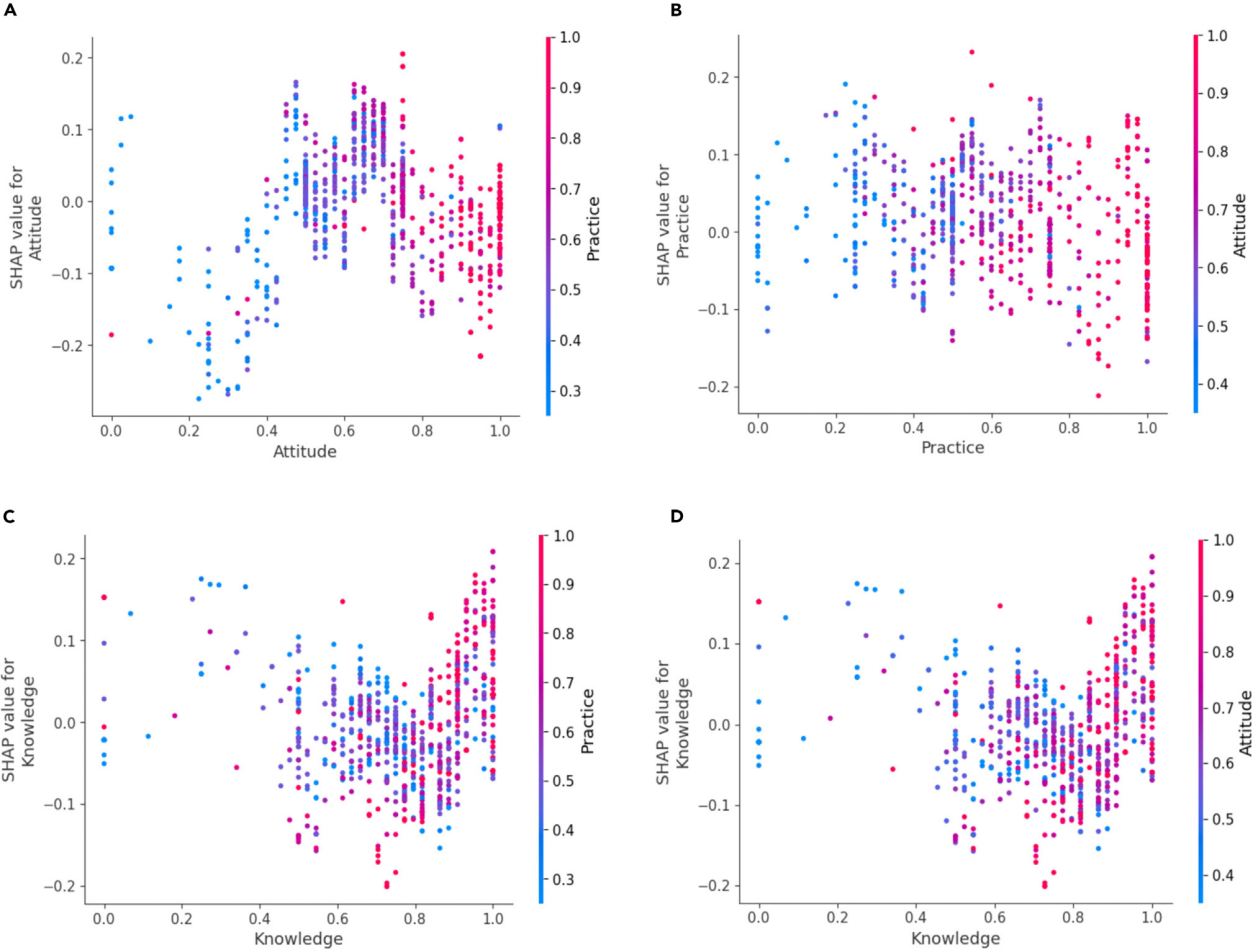
The impact of features on model output (healthy = 1, unhealthy = 0) (A) The relationship among attitude, practice and SHAP values for attitude. (B) The relationship among practice, attitiude and SHAP values for practice. (C) The relationship among knowledge, practice and SHAP values for knowledge. (D) The relationship among knowledge, attitude and SHAP values for knowledge.

The x axis and left y axis in Figures 4C and 4D show that knowledge values have a nonlinear relationship with their SHAP values, and the value of knowledge lies at approximately 0.8, showing a trend shift. Figure 4A shows that attitude values exhibit a nonlinear relationship with their SHAP values, and the value of attitude lies at approximately 0.7, showing a trend shift. Figure 4B shows the relationship of practice values, and their SHAP values are more random in the range of 0–1.

**Figure 5:**
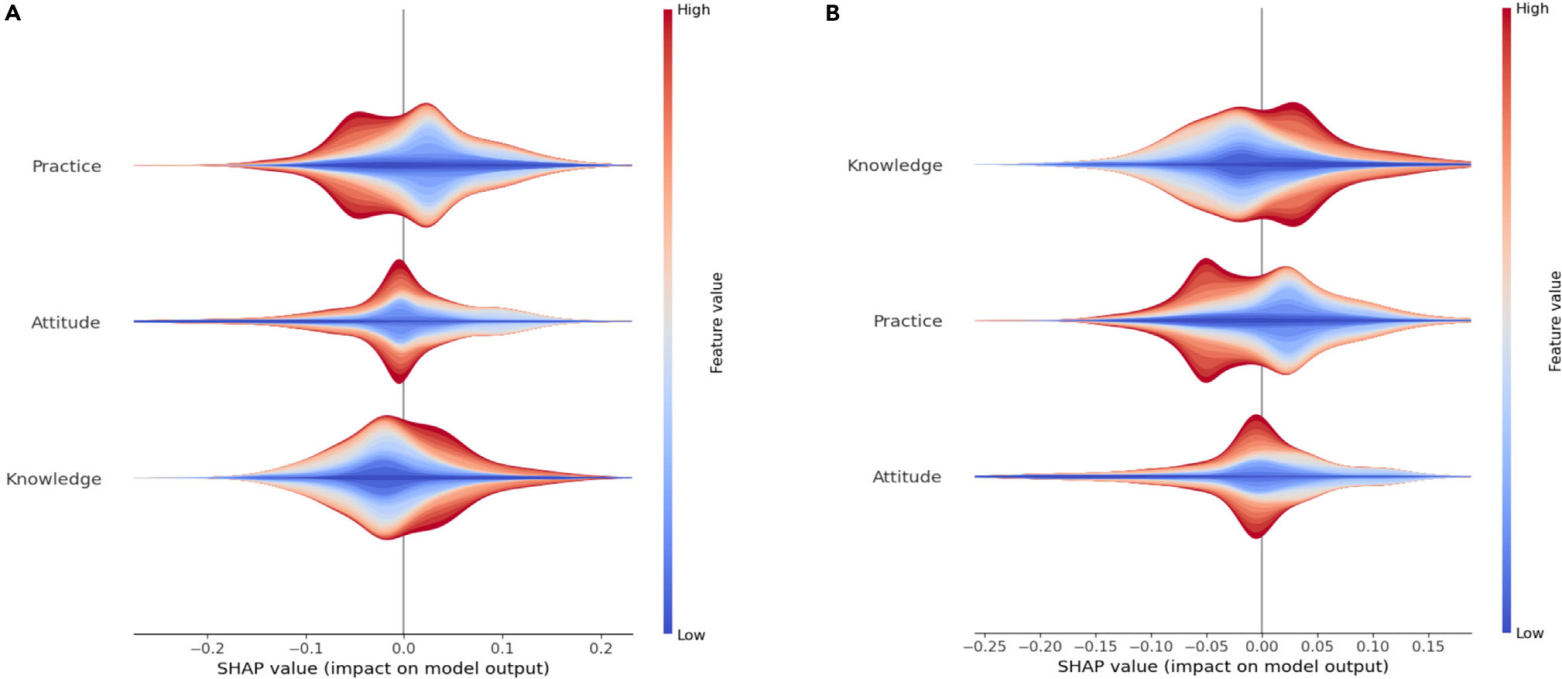
SHAP summary plot related to output in gender group (healthy = 1; unhealthy = 0; A: male; B: female)

According to the x axis and right y axis, Figures 4A–4D show the relationship of each feature, and their relationships are all nonlinear. Figures 5A and 5B show that the higher the attitude, the more likely the practice is to be higher. Figures 4C and 4D show that the higher the knowledge, the more likely the attitude and practice are to be higher.

### Multigroup analysis

As shown in Table 5, the two gender groups were statistically significant in terms of knowledge (p = 0.014), attitude (p = 0.000), and practice (p = 0.000). The city and village groups had statistically significant differences in terms of knowledge (p = 0.004) and practice (p = 0.036), and they had no significant difference in attitude.

**Table 5:** Group differences (independent t test)

	Knowledge			Attitude			Practice		
	Mean	SD	P	Mean	SD	P	Mean	SD	P
Male	78.82	21.459	0.014^∗^	76.46	23.590	0.000^∗∗∗^	72.93	26.039	0.000^∗∗∗^
Female	75.60	18.845		64.65	21.951		58.63	24.650	
City	79.30	21.284	0.004^∗∗^	73.08	11.457	0.138	69.19	27.778	0.036^∗^
Village	75.65	19.551		70.92	10.099		65.79	24.669	

P, probability value: ∗p < 0.05, ∗∗p < 0.01, ∗∗∗p < 0.001; SD: standard deviation.

**Figure 6:**
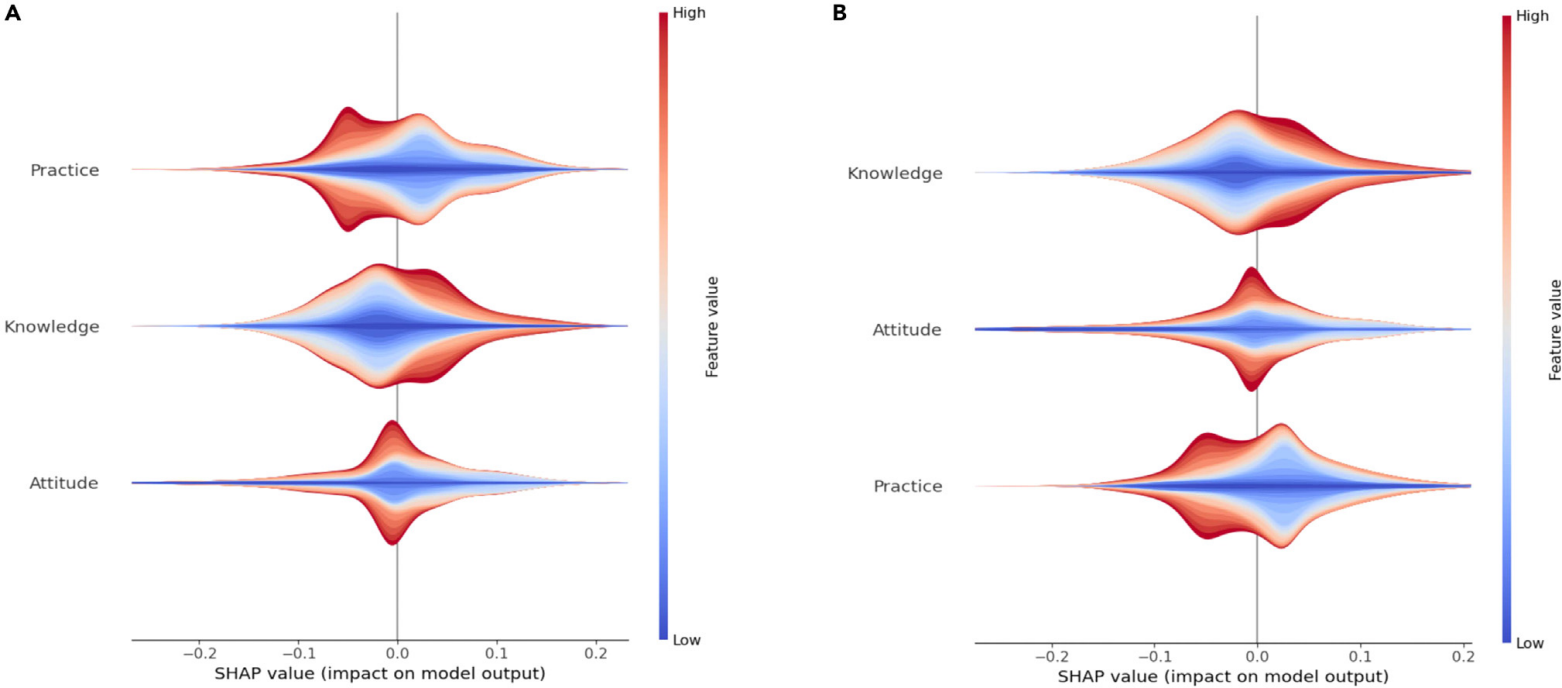
SHAP summary plot related to output in city and village groups (healthy = 1; unhealthy = 0; A: city; B: village)

Figure 5 shows the local analytical results in the gender group. The results in the male group show that practice ranks first, followed by attitude and knowledge, indicating that practice has a strong negative impact on health. The results in the female group show that knowledge ranks top, followed by practice and attitude, indicating that knowledge has a strong positive impact and that practice has a negative impact on health. Figure 6 shows the local analytical results in the gender group. The results in the city group indicate that practice ranks top, followed by knowledge and attitude, which is consistent with the global analytical results. The results in the village group show that knowledge ranks top, followed by attitude and practice.

## Discussion

This study is an inquiry into the effects of campus soccer education activities on young people’s health based on the KAP hypothesis model with an added health component.^22^ It aims to identify the key challenges that have arisen in more than a decade of implementing campus soccer education. The strong correlation between exercise and health has been well established by academic evidence. Hence, it was plausible to include it in the proposed framework. This study confirms that the KAP model adequately explains campus soccer behavior through SEM. Moreover, it uses both SEM and machine learning methods to assess how the knowledge, attitudes, and behaviors acquired by young people through campus soccer education influence their health outcomes from linear and nonlinear perspectives.

This study reveals that soccer knowledge has a significant impact on the attitudes of young people toward soccer, which is consistent with Dishman’s findings.^31^ He discovered that as soccer knowledge increases, so does positive attitude. Meanwhile, Vanttinen et al. contend that better soccer knowledge fosters a favorable outlook on soccer.^32^ This study also demonstrates that attitude influences behavior considerably, in line with previous findings on this topic.^33^ A previous study verified that attitude is a crucial predictor of individual behavior.^33^ Regarding soccer, Schei argues that an optimistic attitude enhances one’s performance and conduct.^34^

Contrary to previous research, this study shows that soccer knowledge does not directly promote positive soccer practice. Fabrigar indicated that individuals with superior soccer knowledge exhibit better soccer practice.^35^ Moreover, higher soccer knowledge implies more involvement in soccer activities.^36^ However, these studies overlooked the effects of attitude. A previous study indicated that knowledge and practice are only weakly linked.^35^ Based on the KAP hypothesis model, individual behavior results from a sequential process of acquiring knowledge, forming beliefs, and acting accordingly. Knowledge may be necessary but not sufficient for practice. Hence, this study employed SEM to examine its mediating effect. The findings revealed that soccer knowledge significantly influences soccer practice through soccer attitude.

This study reveals that soccer knowledge, attitude, and practice all have insignificant impacts on individual health, diverging from previous studies.^37^^,^^38^^,^^39^^,^^40^^,^^41^^,^^42^ Previous studies have shown that individuals with positive attitudes toward sports tend to enjoy better mental health.^40^ Jordan argued that favorable sports attitudes lead to sports participation,^43^ which in turn enhances individual well-being. Moreover, soccer-related activities have been demonstrated to boost physical and mental health effectively.^44^ The researchers attribute this discrepancy to the serious challenges of implementing soccer activities in campus soccer education. This study also investigates three indirect pathways from soccer knowledge to health. The results suggest that soccer knowledge does not improve individual health by fostering positive attitudes toward soccer, nor does it improve well-being by indirectly stimulating soccer engagement. This corroborates the authors’ hypothesis that young people struggle to translate the attitudes and practices acquired from campus soccer education into better health outcomes.

SEM is believed to be the most effective technique for removing any biasing effect caused by measurement errors and building the most appropriate technique for investigating the relationship between observed and latent variables.^45^ However, previous studies have shown that knowledge, attitude, practice, and health may have a nonlinear relationship.^46^ SEM is a suitable approach to test hypotheses with a linear relationship, but it cannot address nonlinear relationships.^45^ To analyze the nonlinear relationships among soccer knowledge, attitude, practice, and health, we selected an optimal machine learning algorithm based on the aforementioned situation. Previous studies have also used SEM and machine learning or deep learning algorithms to examine complex social problems,^47^ which is consistent with our approach. Moreover, we applied the SHAP method to visualize and interpret the global and local importance of each feature. Our results showed that soccer practice was the least important factor for individual health, corroborating the findings from the SEM method.

The main purpose of soccer education and soccer activities in schools is to achieve health promotion through soccer practice.^48^ Therefore, the practice should be a relatively important part of the sustainable development of physical and mental health. Meanwhile, a previous study showed that the health, fitness, and other benefits of soccer participation and practice were well recognized,^49^ which are essential parts to prompt health. However, the results indicate that soccer practice plays a slight role in health conditions in current Chinese soccer education. There are three possible reasons for these results:

(1) Unscientific soccer practice may reduce the importance of practice for health promotion. Many previous studies have shown that exercise and sports participation can indeed serve a health-promoting function,^50^ but the wrong motions, inappropriate amount of exercise, etc., have a negative effect on health. In the past decade, although campus soccer has been strongly promoted by the national government, there is still a serious shortage of professional soccer physical education teachers on campuses, leading to the formation of incorrect technical movements during exercise, which can lead to sports injuries. At the same time, campus soccer is primarily taught through large class teaching, and it is difficult for teachers to pay attention to all students, which may also lead to athletic injuries. Regarding students, previous study has shown that the obesity rate and cardiorespiratory fitness of Chinese students are worsening annually,^51^ and such physical fitness may make it difficult for some students to withstand the load of soccer exercise, which may lead to psychological rejection of students and may have negative effects on mental health. Meanwhile, obese students are more likely to suffer joint injuries during more strenuous sports.^52^

(2) Lower social support may reduce the importance of practice for health promotion. The researchers argue that campus soccer education could enhance soccer knowledge among youths, foster their interest in soccer, and increase their engagement in soccer practices. The frequency and duration of soccer participation significantly affect individual health^53^; however, there is very little social support for joining soccer activities in China,^54^ as society, schools, and families prioritize academic success over everything else. This leads to a substantial gap between the intended and actual outcomes of young people’s involvement in soccer.^55^ Moreover, a top-down social system may have led some leaders to neglect students’ health and lead practices for formalities’ sake in campus soccer education. This misguided approach resulted in many students taking part in soccer practice half-heartedly, hindering the goal of promoting health. This accounts for the upsetting results regarding campus soccer over the past decade.

(3) The lack of sustainability and balance of resource allocation in campus soccer at the various stages of education leads to a situation in which soccer practice is likely to fail to become a lasting behavior. Campus soccer in China’s teaching system often encounters problems such as receiving campus soccer education and practice in primary school but not in schools that move on to middle school.^56^ Meanwhile, a relative study indicated that appropriate sport or practice has a health-promoting effect when it becomes a continuous behavior.^57^ When practice is interrupted or stopped, the health-promoting function is gradually reduced.

Finally, multigroup analysis showed that there were significant differences in the research results between the gender and city vs. village groups. As shown in Table 5, the scores of soccer knowledge, attitude, and practice of males were significantly higher than those of females, which indicated that the male group was more willing to participate in campus soccer activities. In addition, when analyzing the differences between various city vs. village groups, this study found that the scores of soccer knowledge, attitude, and practice of the city groups were higher than those of the village groups, and there are significant differences in their knowledge and practice scores.

In addition, the results in the SHAP local interpretation of the machine learning model found that there are differences in the importance of influencing features on health between males and females. Soccer practice had a greater impact on males’ physical health, while knowledge had a greater impact on females’ health. Previous studies have shown that males are more willing to participate in physical activities and perform risky behaviors to obtain stimulation due to physiological characteristics than females.^58^ However, in the current campus soccer environment, this nature often leads to sports injuries caused by practice, aggravating the negative impact of practice on health. In contrast, females practice less than males, which is consistent with the World Health Organization’s survey results; females’ physical activity level is significantly lower than that of males.^59^ This may reduce the occurrence of injuries and other situations, reducing the negative effects of practice. Previous studies have shown that physical knowledge has a significant impact on physical and mental health,^37^^,^^38^^,^^60^^,^^61^^,^^62^^,^^63^ which may lead to female soccer knowledge of practices or activities outside campus soccer having a health-promoting effect.

In addition, in the analysis of city vs. village students, the study found that the city students showed that practice was the most important feature, while the analysis of the village students showed that practice was the least important feature. This may also reflect the problem of uneven distribution of campus soccer resources. Related studies have shown that China is a typical large country with significant urban-rural differences and uneven regional economic development,^64^ which is considered an important influencing factor for the results of this study. Campus soccer education first started in city areas and then spread to village areas. Campus soccer activities were more frequent in city areas, and village areas had more well-equipped soccer fields, resulting in more soccer participation behaviors among city people. Therefore, at this stage of campus soccer activities, individuals from cities are more likely to have a negative impact on health during soccer participation. In contrast, village people participate less in soccer practice but obtain soccer-related sports nutrition and health knowledge.

In conclusion, this study strongly indicates that the current implementation of campus soccer education has not effectively contributed to the overall health improvement of young students. Despite fostering student participation in soccer, concerns and shortcomings in the implementation process have hindered its ability to promote the physical and mental well-being of adolescents. In essence, the past decade of campus soccer implementation can be considered somewhat unsuccessful. It is essential to address these issues to ensure that sports programs genuinely contribute to the holistic health of youth.

### Limitations of the study

However, since the present study was a cross-sectional study, causality could not be established. In other words, malnutrition may lead to impaired physical functioning, or one’s own health may be the cause of soccer-related knowledge. Also, although this study is a survey of youth across the country, resource constraints and other reasons may result in an uneven distribution of the regions to which the sample belongs. During the long process of teaching soccer in schools, there are some individuals who may not have been adhering to the sport of soccer, which may also affect the results of the study. In addition, the structural equation modeling used in this study may not handle non-linear data well. Therefore, machine learning algorithms with the ability to handle nonlinear data were also used in this study for additional analyses.

## STAR★Methods

### Key resources table

**Table:** 

REAGENT or RESOURCE	SOURCE	IDENTIFIER
**Software and algorithms**		

Python 3.8	Python Software Foundation	https://www.python.org/
IBM SPSS Statistics 26	IBM, Armonk, N.Y., USA	https://www.ibm.com/cn-zh/spss
IBM SPSS AMOS 25	IBM, Armonk, N.Y., USA	https://www.ibm.com/cn-zh/products/structural-equation-modeling-sem

### Resource availability

#### Lead contact

Further information and requests for resources and reagents should be directed to and will be fulfilled by the lead contact, Honglin Song (202221070036@mail.bnu.edu.cn).

#### Materials availability

This study did not generate new unique reagents.

#### Data and code availability

• All data reported in this paper will be shared by the lead contact author upon request.
• This paper does not report original code.
• Any additional information required to reanalyze the data reported in this paper is available from the lead contact upon request.

### Method details

#### Study design

A cross-sectional survey was conducted by using an online self-administered questionnaire. The target group was freshman and sophomore undergraduate students of any age in China who had received campus soccer education and did not have physical ailments that would affect their play. This target group was chosen because, since the publication of China’s campus soccer education policy in 2009, these groups have received nearly 14 years of more complete campus soccer education from primary school to the university phase within the school environment.

#### Sample and data collection

Samples were eligible if they (1) received Chinese soccer education, (2) did not have ailments that would affect their sport, and (3) were freshman and sophomore undergraduates. The sample size was calculated using the population proportion statistical formula^65^: $N=\frac{Z^{2}\times P\left(1–P\right)}{d^{2}}$ (Z = 1.96, p = 65.26%, d = 0.05), where Z = critical value corresponding to 95% confidence level = 1.96; P = proportion with parameter (the proportion of children and adolescents aged 6 to 19 who regularly participate in physical activity is approximately 65.26%, which was from a previous study^66^). Therefore, the calculated sample size was 348, and after considering a 20% nonresponse rate, the minimum required sample size was 436.

Data were collected from December 2022 to January 2023 using the Questionnaire Star Platform, the most frequently used communication and social platform in China. Then, this study distributed the online questionnaire by means of WeChat software. All questions in the questionnaire were had to be completed, and each IP address could be used only once. After data collection, the researchers adopted data cleaning by using Microsoft Excel and IBM SPSS 26. To eliminate invalid data, this study employed two steps:

1) An embedded lie scale was used to exclude 5749 questionnaires that failed to meet the requirements according to three items: “Your grade”, “Participation in campus soccer education or activities”, and “Physical diseases that hinder exercise”; 2) Singular value analysis was applied to detect response patterns and 601 dishonest questionnaires were removed based on their Mahalanobis distance.^67^ A total of 7419 questionnaires were initially collected for this study. After exclusion, there was a total of 1069 valid samples, which met the minimum sample described above.

#### Instruments

To measure knowledge of, attitudes toward, practices involving and health related to Chinese soccer education among participants, the researchers used one study instrument. As shown in Table S1, the KAP scale was developed based on the relevant “Youth training syllabus of Chinese Soccer Association”, “National Youth School Soccer Teaching Guide”, guidelines and previous studies.^68^

The instrument for this study comprises four sections. The first section contains 36 items related to the eight dimensions of health. This scale was developed during the Medical Outcomes Study (MOS) to capture patients’ perceptions of their own health and well-being and has been proven to have high validity and reliability.^69^ The second section contains 11 items related to SK. The SK scale consists of items about soccer story (SS), soccer equipment (EP), soccer rules (SR), nutrition knowledge (NK), and sports health (SH), which were taken from previous studies.^68^^,^^70^ The third section contains 10 items related to SA, which consists of items about emotional attitude (EA), cognitive attitude (CA) and practical attitude (PA). The items from this section were taken from Vlachopoulos.^71^ Finally, the last section contains 10 items related to SP, consisting of items about soccer watching, participation, support and so on, which were also taken from the official guide to campus soccer. All the items were adopted on a 5-point Likert scale, where 1 represented strongly disagree and 5 represented strongly agree.

##### Demographics

Demographic data included gender, grade, location, whether they had received Chinese soccer education, and whether they had ailments that would affect their sport. Below table shows these items.

**Table:** Demographic profile of respondents

Respondents’ characteristics	Item	Frequency (n = 1069)	Percentage (%)
Gender	Male	673	63.0%
	Female	396	37.0%
City vs. village	City	615	57.5%
	Village	454	42.5%
Region	East China	220	20.5%
	North China	55	5.1%
	Central China	87	8.1%
	Southwest China	290	27.0%
	Northwest China	57	5.3%
	Northeast China	301	28.1%

##### Health

The SF-36 version 2 Health Survey is a generic instrument that measures health-related quality of life across eight domains: physical functioning, role-physical, bodily pain, general health, vitality, social functioning, role-emotional and mental health.^72^ It was developed during the MOS to capture patients’ perceptions of their own health and well-being. Using the SF-36v2 to measure health outcomes can assess the impact of exercise on both physical and mental aspects of health thorough soccer education.

#### SEM analytical strategies

This study employed a two-step strategy to test the hypothesized model.^73^ First, to ensure the reliability and validity of the measurement instrument, the researcher invited 5 experts to evaluate the scales and conducted a pilot study by collecting 64 responses from different universities. The questionnaire content was refined, and its reliability and validity were enhanced based on feedback and outcomes. The initial results showed an internal consistency of the constructs ranging between 0.85 and 0.96, which fully complied with the proposed criterion of 0.70–0.90 as a measure of good internal consistency suggested by Terwee et al.^74^ The KMO is 0.826, Bartlett test: χ 2 = 1839.489, df = 406 (p = 0.000), which indicates that confirmatory factor analysis on this model can be conducted.^75^ Considering the initial survey’s results, the researchers initiated the comprehensive survey.

Second, this study validated the measurement model using confirmatory factor analysis and then assessed the fit indices and path coefficients of the hypothesized model using SEM. This study used SPSS 26.0 and AMOS 25.0 for CFA and SEM analysis. Before conducting SEM analysis, we examined the reliability and consistency of the questionnaire data. As shown in table below, all Cronbach’s alpha (a) coefficients exceeded the standard value of 0.6, indicating that the scale was reliable. Confirmatory factor analysis (CFA) is a part of structural equation modeling.^76^ CFA should be completed before analysing structural equation modeling to test structural validity. Structural validity includes convergent validity and discriminant validity. We performed CFA for each dimension. As shown in table below, factor loadings ranged from 0.6 to 0.99, composite reliability (CR) ranged from 0.66 to 0.9, and all extracted average variance (AVE) values ranged from 0.5 to 0.9. All values surpassed the standard values.^77^ Therefore, the questionnaire had appropriate convergent validity. Discriminant validity indicates whether there are significant differences between two dimensions. As shown in table below, diagonal values represent each latent variable’s average variance more than nondiagonal values represent each latent variable’s correlation coefficient, indicating better discriminant validity of scale data.^77^ Therefore, this study’s reliability and validity were good and could be further analyzed.

**Table:** Discriminant validity verification

	Knowledge	Attitude	Practice
Knowledge	0.8226		
Attitude	0.599	0.936	
Practice	0.518	0.933	0.9448
AVE square root	0.906972987	0.96747093	0.97200823

AVE, average variance extracted.

**Table:** Confirmatory factor analysis

Path		Estimate	AVE	CR	Mean	SD	Cronbach’s alpha
Knowledge	SS	0.700	0.8226	0.9581	3.74	1.255	0.930
	SE	0.946			4.19	1.101	
	SR	0.924			4.01	1.184	
	NK	0.978			4.23	0.946	
	SH	0.958			4.28	0.948	
Attitude	EA	0.959	0.9349	0.9773	3.92	1.038	0.977
	CA	0.945			3.99	1.001	
	PA	0.996			3.73	1.101	
Practice	DP	0.971	0.9428	0.9706	3.72	1.128	0.983
	INP	0.971			3.69	1.146	
SS	KQ1	0.642	0.5759	0.7267	3.62	1.342	0.706
	KQ2	0.860			3.85	1.149	
SE	KQ3	0.613	0.6010	0.7438	3.97	1.185	0.706
	KQ4	0.909			4.40	0.963	
SR	KQ5	0.572	0.5064	0.6650	3.88	1.266	0.638
	KQ6	0.828			4.14	1.081	
NK	KQ7	0.935	0.8799	0.9361	4.22	0.952	0.936
	KQ8	0.941			4.24	0.939	
SH	KQ9	0.887	0.8780	0.9557	4.23	0.977	0.955
	KQ10	0.969			4.30	0.933	
	KQ11	0.953			4.30	0.932	
EA	AQ1	0.921	0.8438	0.9555	4.03	0.989	0.949
	AQ2	0.791			3.70	1.121	
	AQ3	0.979			3.95	1.007	
	AQ4	0.971			3.99	0.998	
CA	AQ5	0.957	0.8156	0.9298	3.86	1.068	0.944
	AQ6	0.902			4.03	0.976	
	AQ7	0.847			4.08	0.940	
PA	AQ8	0.963	0.8687	0.9520	3.68	1.113	0.960
	AQ9	0.944			3.69	1.101	
	AQ10	0.926			3.83	1.081	
DP	PQ1	0.922	0.8920	0.9612	3.80	1.103	0.979
	PQ2	0.955			3.76	1.102	
	PQ3	0.963			3.71	1.123	
	PQ4	0.968			3.72	1.126	
	PQ5	0.945			3.60	1.173	
INP	PQ6	0.941	0.8586	0.9681	3.74	1.128	0.969
	PQ7	0.949			3.69	1.153	
	PQ8	0.894			3.65	1.164	
	PQ9	0.937			3.58	1.183	
	PQ10	0.911			3.81	1.085	

SS, soccer story; SE, soccer equipment; SR, soccer rules; NK, nutrition knowledge; SH, sports health; EA, emotional attitude; CA, cognitive attitude; PA, practical attitude; DP, direct practice; INP, indirect practice; KQ_n_, knowledge question n; AQ_n_, attitude question n; PQ_n_, practice question n.

#### Machine learning modeling

Before applying data to models, the study used Min-Max scaling. This study compared five machine learning algorithms: decision tree (DT), random forest (RF), light gradient boosting machine tree (LightGBM), and gradient boosting + categorical features (Catboost). All of them applied a Bayesian optimization algorithm to optimize the hyperparameters based on tree-structured Parzen estimators (TPEs), and the search spaces and final hyperparameters of the four algorithms are shown in Tables S2–S5. In the performance comparison among models, recall, accuracy, precision, and F1 were used in this study, as shown in Equations 1, 2, 3, and 4. Then, this study selected the best algorithm to explain the model globally as well as locally using SHAP, which is consistent with previous studies.^78^^,^^79^^,^^80^ The local interpretations have two perspectives: 1) gender (male, female) and 2) city vs. village. All data processing and modeling were performed via Python 3.8. The whole process of modeling and SHAP analysis as shown in below figure.

**Figure:**
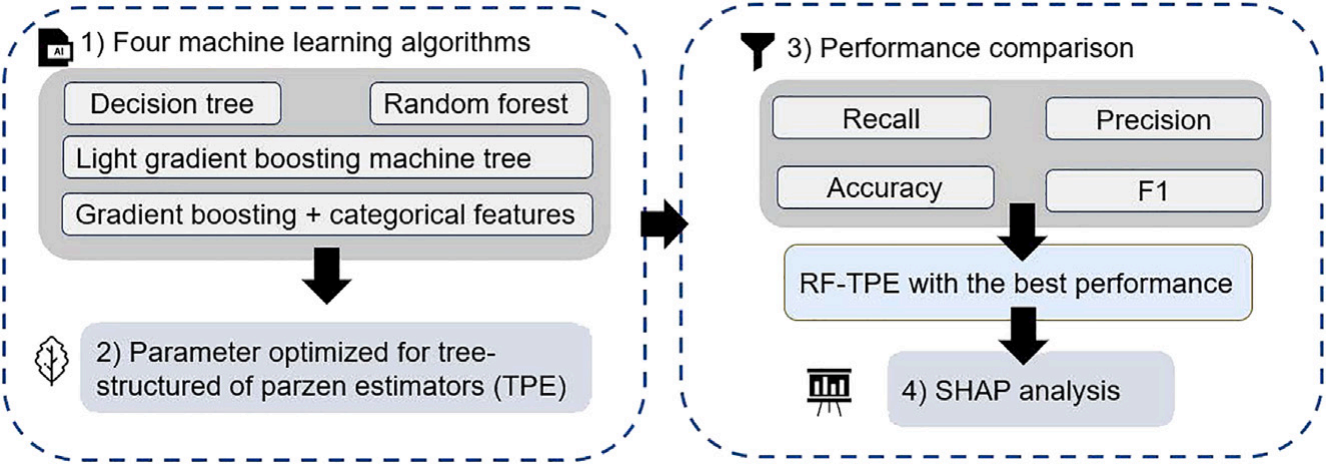
The process of modeling and SHAP analysis

(Equation 1) $$\text{Accuracy}=\frac{\text{TP}+\text{TN}}{\text{TP}+\text{TN}+\text{FP}+\text{FN}}$$

(Equation 2) $$\text{Recall}=\frac{\text{TP}}{\text{TP}+\text{FN}}$$

(Equation 3) $$\text{Precision}=\frac{\text{TP}}{\text{TP}+\text{FP}}$$

(Equation 4) $$F1=\frac{2\times P\times R}{P+R}$$

TP, true & positive; FP, false & positive; FN, false & negative; TN, true & negative.

##### Decision tree (DT)

DT is a non-parametric supervised learning algorithm utilized for both classification and regression tasks. Comprising a hierarchical tree structure, it consists of a root node, branches, internal nodes, and leaf nodes.

##### Random forest (RF)

RF stand out as one of the well-established bagging algorithms recommended by Breiman.^81^ RF is a tree-based algorithm employing decision trees as its base learners. This versatile approach is applicable to both classification and regression tasks.

##### Light gradient boosting machine tree (LightGBM)

The main features of LightGBM are the gradient-based one-side sampling decision tree algorithm, exclusive feature bundling, and a histogram and leaf-wise growth strategy with a depth limit. Gradient-based one-side sampling can strike a good balance between the number of samples and the accuracy of LightGBM’s decision tree.

##### Gradient boosting + categorical features (Catboost)

Catboost is also a member of the gradient boosted decision tree machine learning ensemble techniques. Since its debut in late 2018,^82^ researchers have successfully used CatBoost for machine learning studies.

##### SHAP

SHAP is an approach based on game theory to describe the performance of a machine-learning model. To produce an interpretable model, SHAP uses an additive feature attribution method, i.e., an output model is defined as a linear addition of input variables^83^ and provides a unique output value for a given input indicator.^84^ Based on the additive feature attribution methods, SHAP constructs a linear function g with binary variables to estimate the target function f. The explanation model is as follows^84^:

(Equation 5) $$F\left(x\right)\approx g\left(z^{\prime }\right)=\phi_{0}+\sum_{i=1}^{M}\phi_{i}z_{i}^{\prime }$$

where $z^{\prime }\approx x^{\prime }$, where $x^{\prime }$ is the simplified input mapped from the original inputs $x=h_{x}\left(x^{\prime }\right)$ and ensures that $g\left(z^{\prime }\right)\approx f\left(h_{x}\left(x^{\prime }\right)\right)$; $\phi_{0}$ is the contribution with zero inputs, and M is the number of simplified input features.

##### TPE

This study applies a Bayesian optimization algorithm based on TPE to automatically optimize the models’ hyperparameters. TPE is a sequential model-based optimization (SMBO) algorithm.^85^ The SMBO algorithm can track past evaluation results and decide what to try next, which results in better results at a more minor cost.

### Quantification and statistical analysis

Statistical analyses were performed using Python, IBM SPSS Statistics and AMOS.
