# Supplementary material for: Exploring the relationship among soccer-related knowledge, attitude, practice, and self-health in Chinese campus soccer education
Source: iScience. 2024 Mar 4;27(4):109409. doi: 10.1016/j.isci.2024.109409 (PMC10992701; doi:10.1016/j.isci.2024.109409)
Supplement: Document S1. Tables S1–S5 [file mmc1.pdf]

iScience, Volume *27*

Supplemental information

Exploring the relationship among soccer-related knowledge, attitude, practice, and self-health in Chinese campus soccer education

Honglin Song, Yutao Li, Zhenhang Zhang, and Tianbiao Liu

This file includes:
Supplemental Tables 1-5

Table S1. The soccer-related KAP scale. Related to STAR Methods.

Constructs	Items	Content
Soccer Knowledge	2	Soccer Stories and General Knowledge
	2	Basic knowledge of soccer equipment and field
	2	Soccer competition methods and rules
	2	Nutrition Knowledge
	2	Sports injury management prevention
	3	Technical and tactical principles
Soccer Attitude	4	Emotional Attitude
	3	Cognitive Attitude
	3	Behavioral Attitude
Soccer Behavior	5	Direct participation in sports behavior
	5	Indirect participation in sports behavior
Soccer Story	KQ1	China's men's soccer team reached the World Cup finals for the first time in 2002 in Korea and Japan
	KQ2	The only goal scorer recognized by the FIFA History and Statistics Committee with more than 1,000 goals is King Pele
Basic Equipment	KQ3	Leg pads must be worn in school soccer competitions
	KQ4	For safety reasons and game needs, it is recommended that goalkeepers wear gloves during school soccer competitions
Soccer Rules	KQ5	A soccer player in an offside position is a foul
	KQ6	Two yellow cards in a soccer match become one red card
Nutrition Knowledge	KQ7	The adverse effects of dehydration can be reduced through proper rehydration and sugar supplementation
	KQ8	Soccer players to consume more protein in their diet, but not in excess
Sports Health	KQ9	Soccer program is dangerous, should have more knowledge of rehabilitation
	KQ10	Soccer players have higher rates of ankle and knee injuries
	KQ11	High-load soccer sports on an empty stomach and lack of sleep can easily produce sports injuries
Emotional Attitude	AQ1	I like soccer class
	AQ2	I sulk when a soccer event is cancelled for some reason
	AQ3	Playing soccer makes me feel better
	AQ4	Every time you play soccer, you have a new experience and root new feelings, you can feel the fun of sports
Cognitive Attitude	AQ5	I love soccer and the culture is very attractive to me
	AQ6	I think soccer is a good recreational activity
	AQ7	Playing soccer is good for yourself, your family and your country
Practice Attitude	AQ8	I wish I could play soccer every day
	AQ9	I will convince people around me to play soccer with me
	AQ10	See someone playing soccer I also want to play soccer
Direct Practice	PQ1	I will take the initiative to participate in soccer activities of off-campus soccer organizations
	PQ2	I can tell if I am in an offside position in a soccer game based on the game situation
	PQ3	I can effectively organize an attack during a soccer game
	PQ4	I can play effective defense during soccer games
	PQ5	I am good at using "two-over-one" tactics
Indirect Practice	PQ6	I will actively follow the soccer news
	PQ7	I follow the five major league matches

PQ8	I will watch the soccer league live
PQ9	I will watch soccer magazines
PQ10	I will watch school soccer or games

Table S2. The searching space and optimized hyper-parameters of RF. Related to STAR Methods.

Parameters	Space	Optimized
n_estimators	[500, 510, 520, ···, 2500]	1780
min_samples_split	[2, 3, 4, 5]	5
max_depth	[1, 2, 3, ···, 10]	10

Table S3. The searching space and optimized hyperparameters of LightGBM. Related to STAR Methods.

Parameters	Space	Optimized
n_estimators	[500, 510, 520, ···, 2500]	1790
min_child_samples	[1, 2, 3, ···, 50]	10
num_leaves	[1, 2, 3, ···, 50]	49
depth	[1, 2, 3, ···, 10]	1
learning_rate	[0.001, 0.002, 0.003, ···, 1]	0.636

Table S4. The searching space and optimized hyperparameters of DT. Related to STAR Methods.

Parameters	Space	Optimized
min_samples_split	[2, 3, 4, 5]	4
max_depth	[1, 2, 3, ···, 10]	5

Table S5. The searching space and optimized hyperparameters of Catboost. Related to STAR Methods.

Parameters	Space	Optimized
iterations	[100, 200, 300, ···, 4000]	3200
depth	[1, 2, 3, ···, 10]	1
learning_rate	[0.001, 0.002, 0.003, ···, 1]	0.759
border_count	[1, 2, 3, ···, 222]	136
l2_leaf_reg	[0, 1, 2, 3, ···, 10]	9
